# Traditional Chinese medicine Dingkun pill to increase fertility in women with a thin endometrium—a prospective randomized study

**DOI:** 10.3389/fendo.2023.1168175

**Published:** 2023-09-28

**Authors:** Fengyu Jin, Xiangyan Ruan, Shuang Qin, Xin Xu, Yu Yang, Muqing Gu, Yanqiu Li, Jiaojiao Cheng, Juan Du, Xiaodan Yin, Alfred O. Mueck

**Affiliations:** ^1^ Department of Gynecological Endocrinology, Beijing Obstetrics and Gynecology Hospital, Capital Medical University. Beijing Maternal and Child Health Care Hospital, Beijing, China; ^2^ Department of Traditional Chinese Medicine, Beijing Obstetrics and Gynecology Hospital, Capital Medical University, Beijing Maternal and Child Health Care Hospital, Beijing, China; ^3^ Department of Women’s Health, University of Tuebingen, University Women’s Hospital and Research Centre for Women’s Health, Tuebingen, Germany

**Keywords:** infertility, thin endometrium, Dingkun pill, estrogen, traditional Chinese medicine

## Abstract

**Objective:**

The aim of this study is to optimize the treatment methods of infertility, which is suggested to be mainly caused by thin endometrium, using a special form of traditional Chinese medicine, the Dingkun pill (DKP), to increase the beneficial endometrial effect of conventional hormone/progestogen therapy.

**Methods:**

A total of 307 patients visiting our specialized gynecological endocrinology department because of infertility, which we suggested to be caused by thin endometrium [endometrial thickness (EMT) < 7 mm], were randomly assigned to the experimental group and the control group. The experimental group was treated with estradiol + sequential dydrogesterone + DKP (every day); the control group received hormonal treatment without the Chinese medicine. All patients were monitored in terms of follicle diameter, EMT, and endometrial type every 2 days from the 8th to the 10th day of the menstrual cycle until ovulation day during three menstrual cycles. Serum progesterone levels on 7–8 days after ovulation were measured, and the cumulative pregnancy rate during three menstrual cycles between the two groups was compared.

**Results:**

EMT on ovulation day in the experimental group was significantly higher than that in the control group (7.88 vs. 7.15 mm; *p *< 0.001). The proportion of type A and type B endometrium in total was significantly higher in the experimental group than that in the control group (83.2% vs. 77.7%; *p *< 0.05). Progesterone levels were significantly higher in the experimental group than those in the control group (10.874 vs. 10.074 ng/mL; *p *< 0.001). The cumulative pregnancy rate, the main outcome of the study, was significantly higher in the experimental group than that in the control group (29.2% vs. 15.7%; *p *< 0.05).

**Conclusion:**

DKP added to conventional estrogen/progestogen therapy can significantly improve EMT and luteal function in patients attending due to infertility. Because this regimen increased the cumulative pregnancy rate in our study, we conclude that DKP can be used to increase the so-called “thin endometrium infertility”.

## 1 Introduction

In recent years, the incidence of female infertility has significantly increased due to various factors. It is reported that approximately 8%–12% of couples worldwide and 15% of couples in China are affected by infertility (1, 2). Effectively improving clinical pregnancy will benefit many patients, especially those who regard childbearing as a life mission.

The appropriate endometrium thickness (EMT) is an important single etiology of possible infertility (3). If the EMT is not high enough during ovulation, there is usually a poor reproductive outcome. Although there is no consensus regarding the cutoff value of EMT during ovulation, many studies have shown that the pregnancy rate will decrease significantly if the EMT measured by transvaginal ultrasound is less than 7 mm on the day of hCG or luteal support in assisted reproductive technology (ART) (4–6). Therefore, endometrium with an EMT of less than 7 mm during ovulation is considered thin, and the resulting infertility is regarded as “thin endometrium infertility”.

Proliferation of the endometrium depends on the secretion of estrogen under normal physiological conditions. Hence, there is no doubt that estrogen supplementation is the first choice for thin endometrium infertility. In most cases, estrogen supplementation can actually promote endometrium proliferation, i.e., increase EMT and consequently improve the clinical pregnancy rate (7, 8). However, it is not effective for all patients, and some patients showed low response or no response to estrogen no matter whether it was used in the stimulating cycle, natural cycle, or high-dose hormone supplement cycle (9). The use of high-dose estrogen is limited due to the risk of hyperplasia, which could lead to endometrial cancer. Monitoring by assessing estradiol (E2) levels is difficult, because it has been shown that E2 levels were only correlated with endometrium thickness in patients with relatively low estrogen levels. Once E2 levels are >1,000 ng/mL, it has no further significant impact on endometrium thickness (3, 10). Therefore, it is necessary to explore another effective alternative method to improve EMT and pregnancy rates for those patients with less or even no response to estrogen supplement.

As the exclusive tribute medicine for the imperial palace of the Qing Dynasty, the effectiveness of Dingkun pill (DKP) on hypomenorrhea and female infertility has been repeatedly verified in China (11, 12). In this study, the main endpoint was EMT. The type of endometrium, progesterone (P), and cumulative pregnancy rate were assessed as secondary endpoints to verify the cooperation effect of DPK combined with estrogen in treating thin endometrium infertility.

## 2 Materials and methods

### 2.1 Ethical approval

This study was approved by the ethics committee of Beijing Obstetrics and Gynecology Hospital, Capital Medical University, China (Protocol number: 2018-KY-058-04). All patients provided their informed, signed consent.

### 2.2 Design and participants

**Figure 1:**
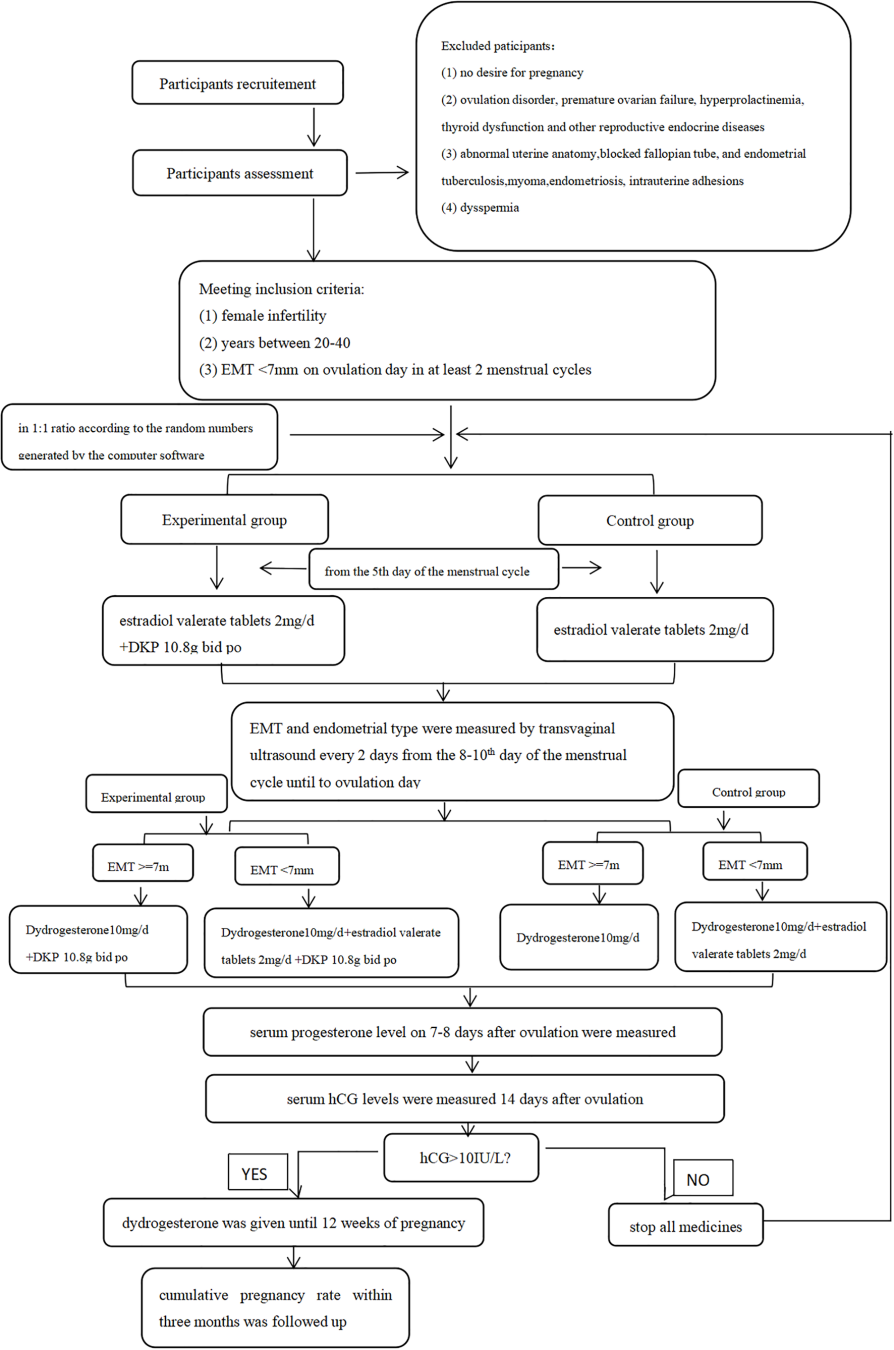
The flawchart.

This study was a prospective, randomized controlled study and the flowchart is shown in **Figure 1**. Patients attending the Department of Gynecological Endocrinology, Beijing Obstetrics and Gynecology Hospital, Capital Medical University between November 2018 and October 2022 were recruited. All patients who met inclusion criteria and exclusion criteria were randomly assigned to the control group or the experimental group in a 1:1 ratio according to the random numbers generated by the computer software and received different treatment schemes. The whole intervention process lasted three menstrual cycles. All patients were natural cycles and received treatments in the outpatient department. Since our research was not focused on evaluating the endometrial receptivity, we do not have sufficient evidence to consider if endometrial biopsies are essential or not. Thus, endometrial biopsy and/or hysteroscopy were only performed on patients who need further diagnostic procedures such as excluding polyps as reasons for bleeding problems.Inclusion criteria were as follows: (I) female infertility, (II) women aged between 20 and 40 years, and (III) EMT less than 7 mm on ovulation day in at least two natural menstrual cycles. Infertility was defined by failure to achieve a successful pregnancy after 12 months or more of appropriate, timed unprotected intercourse or therapeutic donor insemination (13).

Exclusion criteria were as follows: (I) no desire for pregnancy; (II) ovulation disorder, premature ovarian failure, hyperprolactinemia, thyroid dysfunction, and other reproductive endocrine diseases; (III) abnormal uterine anatomy, blocked fallopian tube, endometrial tuberculosis, myoma, endometriosis, and intrauterine adhesions; and (IV) dyspermia.

### 2.3 Measure parameters

#### 2.3.1 Recruitment for the study

Serum endocrine hormones including follicle-stimulating hormone (FSH), luteinizing hormone (LH), and estradiol (E2) were measured on days 2–4 of the menstrual cycle. EMT and endometrium type on ovulation day were also measured by transvaginal ultrasound. The day when the dominant follicle (≥18 mm) disappeared was defined as ovulation day. Endometrium with an EMT of less than 7 mm on ovulation day was considered thin. Only these patients were recruited for the study, i.e., randomly selected for the experimental group or for the control group.

#### 2.3.2 After randomized allocation to the two groups

All patients were monitored in terms of follicle diameters, EMT, and endometrial type every 2 days from the 8th day to the 10th day of the menstrual cycle until ovulation day during three menstrual cycles. Serum progesterone level on 7–8 days after ovulation were also measured. Cumulative pregnancy rate within 3 months was followed up.

#### 2.3.3 EMT and type assay

After the uterus was scanned by the longitudinal section and finding out that the endometrial image was the clearest, the endometrium thickness was determined by measuring the maximum anteroposterior distance between the myometrial and the endometrium interface. The endometrial pattern was assessed according to the classification proposed by Gonen (14)as follows: Type A is a multilayered endometrium consisting of prominent outer and midline hyperechogenic lines and inner hypoechogenic regions. Type B is characterized by the same reflectivity of ultrasound as the myometrium, with a non-prominent or absent central echogenic line. Type C is an entirely homogenous, hyperechogenic endometrium.

#### 2.3.4 Cumulative pregnancy rate

We considered patients pregnant if serum hCG levels were more than 10 IU/L. Cumulative pregnancy rate: total number of pregnancies/total number of patients×100%.

### 2.4 Treatment in the two groups

#### 2.4.1 Control group

The women were treated with estradiol valerate tablets (Bayer, Guangzhou, China) 2 mg/day orally starting on the fifth day of the menstrual cycle. If the EMT was measured by transvaginal ultrasound on ovulation day > 7 mm, the intake of estradiol valerate tablets was stopped, and only dydrogesterone 10 mg/day orally (Abbott Biologicals B.V., Netherlands) was used. If EMT on ovulation day remained< 7 mm, the treatment with estradiol valerate tablets was continued and, at the same time, dydrogesterone 10 mg/day orally was added (so-called “sequential hormone replacement therapy”).

#### 2.4.2 Experimental group

These women received DKP (Luwang, Jilin, China) 10.8 g twice a day orally from the fifth day of the menstrual cycle until 14 days after ovulation. The other drug regimen (hormonal treatment) was the same as that in the control group.

DKP is mainly composed of the following 30 medicinal herbs: Radix Ginseng, Cornu Cervi Pantotrichum, Radix Angelicae sinensis, Radix Rehmanniae Preparata, Stigma Croci, Caulis Spatholobi, Radix Notoginseng, Radix Paeoniae Alba, Rhizoma Atractylodis Macrocephalae, Fructus Lycii, Radix Scutellariae, Rhizoma Cyperi, Fructus Leonuri, Rhizoma Ligustici Chuanxiong, Cornu Cervi Degelatinatum, Colla Corii Asini, Rhizoma Corydalis, Flos Carthami, Herba Leonuri, Faeces Togopteri, Poria, Radix Bupleuri, Radix Linderae, Fructus Amomi Villosi, Cortex Eucommiae, Rhizoma Zingiberis, Herba Asari, Radix Cyathulae, Cortex Cinnamomi, and Radix Glycyrrhizae. The “Dingkun pill” standard is approved by the China Food and Drug Administration (CFDA).

Serum hCG levels were measured in both groups 14 days after ovulation. If hCG >10 IU/L, estradiol valerate tablets were stopped and only dydrogesterone was given until 12 weeks of pregnancy. If hCG<10 IU/L (i.e., no pregnancy), the study was continued accordingly; i.e., treatments were continued on the 5th day of the next menstrual cycle until patients became pregnant or until the end of the third cycle at the end of the study.

### 25 Statistical analysis

#### 2.5.1 Sample size calculation and statistical analysis

We used the following formula for calculating the minimal sample size for this type of study:


$$N={\left[\frac{Z\alpha \sqrt{\pi_{c}(1-\pi_{c})(1/Q_{1}+1/Q_{2})}+Z_{\beta }\sqrt{\pi_{1}(1-\pi_{1})/Q_{1}+\pi_{2}(1-\pi_{2})/Q_{2}}}{\delta }\right]}^{2}$$

Previous research estimated that the clinical pregnancy rate of infertile women with thin endometrium after estrogen treatment was approximately 10%, and the clinical pregnancy rate of other similar TCM was approximately 20% (15). The statistical significance level was set at 5% (α = 0.05) using a one-sided test and 80% power (1 − β), Q1 = Q2 = 0.5, π1 = 20%, π2 = 10%, πc = Q1π1 + Q2π2 = 0.5 * 0.20 + 0.5 * 0.1 = 0.15, δ = π1 − π2 = 10%. We calculated that the minimum sample size should be about *n* = 300.

SPSS software 16.0 (SPSS Inc., Chicago, IL) was used to statistically describe and analyze the research results. The quantitative variables were described by mean ± standard deviation (SD) or median ± interquartile range (IQR). The qualitative variables were described by frequency and percentage. Two-sample *t*-tests were used to compare means. *χ* ^2^ tests were used to compare the frequency. A two-sided *p*-value of<0.05 was considered to indicate statistical significance.

## 3 Results

### 3.1 Clinical characteristics of the study population

We recruited 340 patients in total and 307 patients finished this study, including 154 patients in the experimental group and 153 patients in the control group. A total of 33 patients were lost to follow-up, namely, 17 patients in the experiment group and 16 patients in the control group. The baseline characteristics of all patients are shown in **Table 1**. There was no significant difference between the two groups regarding the baseline parameters including age, BMI, EMT/type on ovulation day, and the serum endocrine hormone levels measured on the 2nd to 4th day of menstruation.

**Table 1:** Basic statistical characteristics of the two groups of patients.

Variables	Experimental group (*n* = 154)	Control group (*n* = 153)	*p*-values
Age (years, $\bar{\chi }$ ± SD)	30.7 ± 4.09	30.9 ± 3.99	0.623
BMI	22.72 ± 2.482	22.45 ± 2.550	0.362
EMT (mm)	5.67 ± 0.83	5.71 ± 0.77	0.717
Count of type A and B Endometrium, *n* (%)	115 (74.7%)	109 (71.2%)	0.532
FSH (mIU/mL)	5.94 ± 1.41	5.80 ± 1.32	0.376
LH (mIU/mL)	6.29 ± 1.84	6.20 ± 1.73	0.665
E2 (pg/mL)	39.056 ± 11.016	38.167 ± 11.515	0.490

BMI, body mass index; EMT, endometrial thickness; FSH, follicle-stimulating hormone; LH, luteinizing hormone; E2, estradiol.

### 3.2 Comparison of endometrium thickness/type and P levels between two groups after treatment

**Figure 2:**
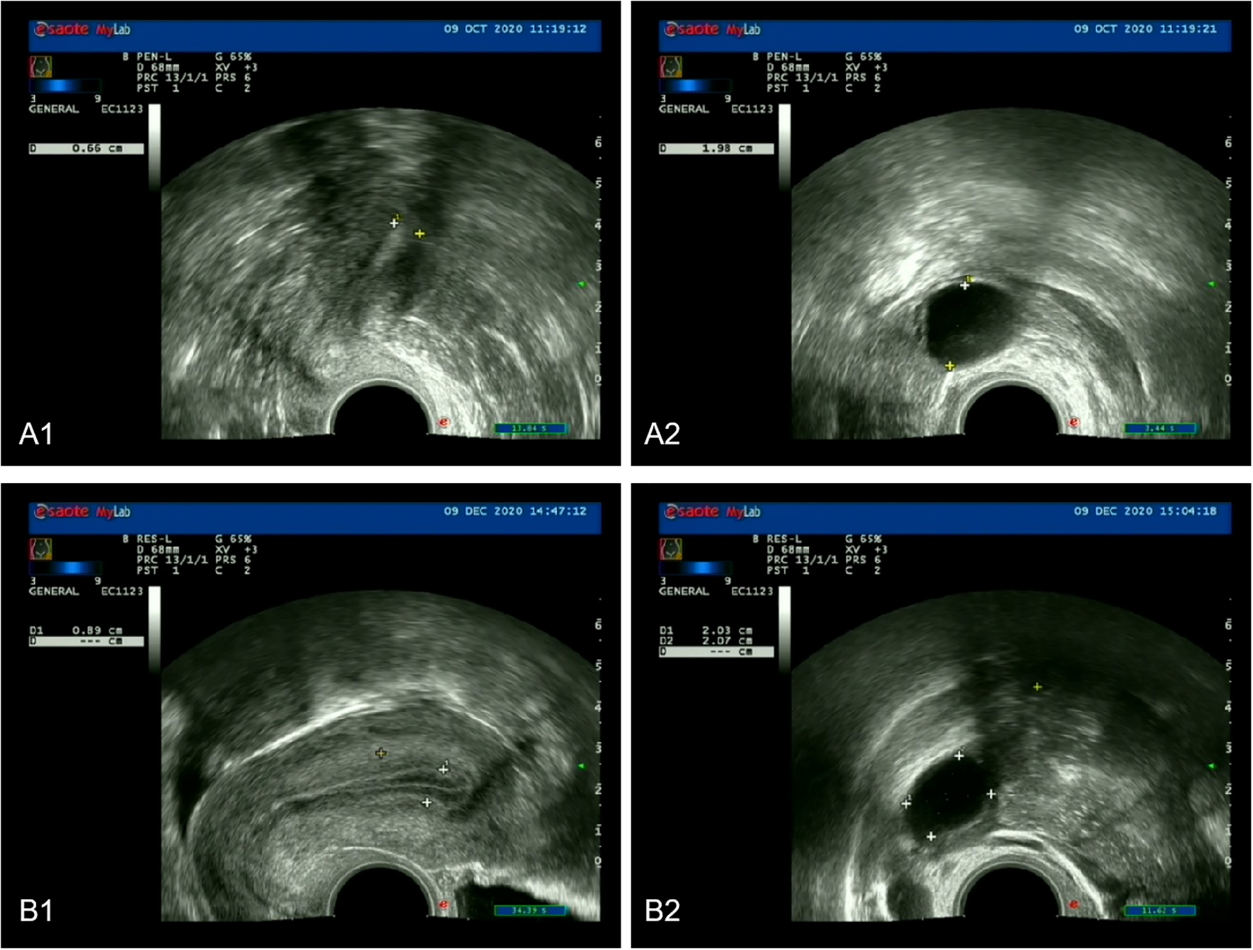
**(A1, A2)** Endometrium and follicle diameter during ovulation before treatments in experimental group. **(B1, B2)** Endometrium and follicle diameter during ovulation after treatments in the experimental group.

As shown in **Table 2** and **Figure 2**, after three menstrual treatments, the average EMT of patients on the ovulation day in the control group and experimental group increased significantly, reaching 7.15 ± 1.40 mm and 7.88 ± 1.37 mm, respectively. The average EMT of the experimental group was significantly higher than that of the control group, with a statistically significant difference (*p*< 0.001). The proportion of type A and type B endometrium in total in the experimental group was significantly higher than that in the control group (83.2% vs. 77.7%; *p*< 0.05). In addition, the serum P level in the experimental group and control group was 10.874 ± 1.723 ng/mL and 10.074 ± 2.130 ng/mL, respectively. The serum P level in the experimental group was significantly higher than that in the control group, (*p*< 0.001).

**Table 2:** Endometrium thickness/type and *p* level on ovulation for 7–8 days of the two groups.

Variable	Group		*p*-values
	Experimental	Control	
EMT (mm)	7.88 ± 1.37	7.15 ± 1.40	0.00***
P (ng/mL)	10.874 ± 1.723	10.074 ± 2.130	0.00***
Count of type A and B Endometrium, *n* (%)	347 (83.2%)	338 (77.7%)	0.047*

***p< 0.001, *p< 0.05; P, progesterone.

### 3.3 Cumulative pregnancy rate

As shown in **Table 3**, the cumulative pregnancy rate was 29.2% (45 of 154 patients) in the experimental group vs 15.7% (24 of 153 patients) in the control group. The cumulative pregnancy rate in the experimental group was significantly higher than that in the control group, with statistical significance.

**Table 3:** Cumulative pregnancy rate between the two groups after 3 months.

Pregnancy	Group		*p*-values
	Experimental	Control	
Yes	45	24	
No	109	129	
Total	154	153	0.005**

**p< 0.01.

### 3.4 Safety and tolerability

All participants rated the tolerability as good. No adverse events were associated with using DKP.

## 4 Discussion

In our study, we found that the average EMT of patients in the experimental group was significantly higher than that in the control group, exceeding the minimum threshold for pregnancy when the same dose of estrogen was supplemented. In contrast, the average EMT of patients in the control group on average just reached the pregnancy threshold, which means that, for some patients, the endometrium proliferating effect was perhaps not sufficient enough for an implantation during a possible pregnancy. Undoubtedly, proper EMT is crucial for embryo implantation. Once the EMT increased in those patients with thin endometrium infertility, the clinical pregnancy rate also increased significantly (16–18). The use of extended estrogen supplementation has solved the problem in some patients, but there were also risks that cannot be ignored. For example, the excessive use of estrogen during the early follicular phase will inhibit the production of follicle-stimulating hormone, affect the quality of oocytes, and increase the risk of endometrial hyperplasia and endometrial cancer. Short-term treatment can also increase the risk of venous thromboembolism. If the EMT can be effectively increased by using other treatment concepts without increasing the dose of estrogen, this will benefit many patients. In fact, DKP showed promising possibilities based on our research.

It was suggested that the mechanisms of DKP in treating infertility may be not only to increase EMT, but also to promote some changes that are beneficial for endometrial receptivity. Impaired endometrial receptivity may account for up to two-thirds of implantation failures in younger women (19). Our results also showed that the level of progesterone and the proportion of type A and B endometrium in the experimental group were significantly higher than those in the control group. These all can have a very favorable impact on endometrial receptivity and embryo implantation, which could be the reason that we achieved a significant increase in pregnancy rate in the experimental group in our study. Because our research was not focused on evaluating endometrial receptivity, we only performed endometrial biopsies for further diagnostics, for example, of unclear bleedings, not to assess endometrial receptivity. However, the improvement of EMT, corpus luteum function, and endometrial blood flow implies an increase in endometrial receptivity in many studies (19, 20). Furthermore, in relevant studies, DKP showed more complex potential mechanisms. DKP not only could increase E2 levels on the day of frozen–thawed embryo transfer and the sensitivity to estrogen during the peri-implantation stage (21, 22), but also could increase numbers of oocytes retrieved, high-quality embryos, embryo implantation rate, and clinical pregnancy rate (23). Although the detailed molecular mechanisms of the effect of DKP on infertility are still unclear, traditional Chinese medicine theory provides a certain explanation. In traditional Chinese medicine theory, kidney function contributes to reproduction mechanisms, and infertility can be caused by kidney deficiency, liver depression, and blood stasis. In fact, modern pharmacology and clinical studies have confirmed that Chinese medicine with the effect of recuperating the kidney function and promoting blood circulation can indeed promote the proliferation of uterine glands and blood vessels in experimental animals, improve uterine artery perfusion, improve pelvic microcirculation, improve endometrial receptivity, and increase the clinical pregnancy rate in infertile patients (24–26). DKP is one of the most representative medicines; it contains Atractylodis Macrocephalae Rhizoma, Poria, Chuanxiong Rhizome, and Angelicae Sinensis Radix, which can recuperate the kidney function, and Leonuri Herba, Corydalis Rhizoma, and Angelicae Sinensis Radix, which can nourish blood and regulate menstruation. DKP alone has proved its effectiveness in treating hypomenorrhea and infertility in many traditional Chinese medicine studies (27). However, until now, there is a lack of studies that investigate the use of DKP combined with estrogen for “thin endometrium infertility”.

There are many factors that can cause infertility, and some are not very clear even. We focused on endometrial factors, but within our diagnostic and clinical procedures, we tried to exclude most of the other common potential causes, including ovulation disorder, premature ovarian failure, hyperprolactinemia, thyroid dysfunction and other reproductive endocrine diseases, abnormal uterine anatomy, blocked fallopian tube, endometrial tuberculosis, myoma, endometriosis, intrauterine adhesions, and dyspermia.

It can be stated that a strength of this study is that all EMT measurements, the main endpoint of this study, were performed by one experienced doctor. The sample size corresponds to the statistical calculation. The study design is prospective and clearly suitable to test the efficacy of DKP under routinely clinical conditions. Only patients with thin endometrium have been included, as a precondition for randomization to the two groups.

The weakness of this trial is that there was no placebo–control due to the limited clinical conditions. Only one dose of estrogen supplement (2 mg per day) was studied. It remains unclear if the use of Dingkun alone, without addition of hormone therapy, would have similar beneficial results. Despite this question being of interest (to reduce hormone-dependent risks such as venous thrombosis), hormonal treatment is the “gold standard” for increasing endometrial thickness in women who wish to conceive. Therefore, our aim was to investigate whether the endometrium proliferating effect of hormones could be further increased by adding this form of traditional Chinese medicine, but without increasing the dosage of the estrogen. Further studies should investigate if the dose of hormones could be decreased, or if hormones could even be completely omitted.

## 5 Conclusion

DKP added to conventional estrogen/progestogen therapy can significantly improve EMT and luteal function in patients attending due to infertility with a thin endometrium. Because this regimen did increase the cumulative pregnancy rate in our study, we conclude that DKP can be used to increase the so-called “thin endometrium infertility”.

## Data availability statement

The original contributions presented in the study are included in the article/supplementary material. Further inquiries can be directed to the corresponding author.

## Ethics statement

The studies involving humans were approved by the ethics committee of Beijing Obstetrics and Gynecology Hospital, Capital Medical University, China (Protocol number: 2018-KY-058-04). The studies were conducted in accordance with the local legislation and institutional requirements. The participants provided their written informed consent to participate in this study.

## Author contributions

All authors qualify for authorship by contributing substantially to this article. FJ: article preparation and study implementation. XR: interpreted the results, provided critical comments, and revised the first draft. SQ, XX, YY, MG, YL, JC, JD, and XY: follow-up. AM: experimental supervision, interpretation of results, and article revision. All authors contributed to the article and approved the submitted version.
